# Post-Lockdown Effects on Students’ Mental Health in Romania: Perceived Stress, Missing Daily Social Interactions, and Boredom Proneness

**DOI:** 10.3390/ijerph18168599

**Published:** 2021-08-14

**Authors:** Liliana Dumitrache, Elena Stănculescu, Mariana Nae, Daniela Dumbrăveanu, Gabriel Simion, Ana Maria Taloș, Alina Mareci

**Affiliations:** 1 Faculty of Geography, University of Bucharest, 010041 Bucharest, Romania; mariana.nae@geo.unibuc.ro (M.N.); daniela.dumbraveanu@geo.unibuc.ro (D.D.); gabriel.simion@geo.unibuc.ro (G.S.); ana.talos@geo.unibuc.ro (A.M.T.); alina.mareci@unibuc.ro (A.M.); 2 Faculty of Psychology and Educational Sciences, University of Bucharest, 050663 Bucharest, Romania; 3 Romanian Young Academy, University of Bucharest, 050663 Bucharest, Romania

**Keywords:** mental health, perceived stress, university students, gender differences, COVID-19, post-lockdown, Romania

## Abstract

The rapid spread of COVID-19 worldwide was accompanied by intense fears, confusion, worries, anger, and stress threatening people’s mental health. Unprecedented measures to slow down and prevent the transmission of COVID-19 have had various impacts on the population’s health behaviour and mental health. The main purpose of the present study is to investigate the lockdown’s effects on university students’ mental health in Romania. Based on a cross-sectional design, the survey data were collected from a sample of 722 participants (247 males; M = 21.1 years; SD ± 1.73). A path analysis was performed to verify the hypothesised direct and indirect effects included in the multiple mediation model. The findings showed a positive association between stress and boredom proneness, missing daily social interactions, spending more time on phone conversations, and the increasing interest in following news about the pandemic. The path analysis revealed an excellent fit between the proposed multiple mediation model and the sample data. Boredom proneness and missing daily social interactions both affected stress, directly and indirectly, through more time spent on phone conversations. In addition, it was found that the increased interest in following news about the pandemic mediated the relationship between boredom proneness and perceived stress. In terms of gender differences, our findings revealed that female students experienced significantly higher stress levels than male students, perceived to a greater extent the lack of daily social interactions, and spent more time on phone conversations. Overall, the findings further extend the empirical evidence on university students’ mental health in the context of the COVID-19 pandemic. Therefore, universities need to organise support programmes focused on developing university students’ coping strategies to maintain their mental health even in adverse contexts.

## 1. Introduction

The crisis due to the emergence of the novel coronavirus (SARS-CoV-2) and the unprecedented lockdowns have had a dramatic impact on the mental health of the world’s population. The World Health Organisation considers the consequences of COVID-19 on the mental health and psychological well-being of the population to be very important [1] and has been warning governments to be prepared to tackle mental health complications. 

Even though the approach and response to quarantine, social distancing, and isolation have differed notably across populations, it has been unanimously acknowledged that restrictive measures increased anxiety and fears and affected different populations’ reactions while facing these stressful situations. National surveys undertaken during the initial stages of the pandemic revealed that a third or more of the adult population were distressed. The way the disease itself spread and the strict control measures, such as nationwide lockdowns, social distancing, and isolation, that had to be implemented by countries from March 2020 onwards, can have long-lasting adverse effects on the mental health of the various populations who have been suffering through all this. These adverse effects could include acute panic, fear, anxiety, obsessive behaviours, depression, and post-traumatic stress symptoms [2,3,4].

Xiong et al. [5] found relatively high rates of symptoms of anxiety (6.33–50.9%), depression (14.6–48.3%), post-traumatic stress disorder (PTSD) (7–53.8%), psychological distress (34.43–38%), and stress (8.1–81.9%) among the general population during the COVID-19 pandemic in China, Spain, Italy, Iran, the US, Turkey, Nepal, and Denmark, with the most exposed being females, the younger age group, and students.

Prati et al. [6] conducted a review and meta-analysis of 25 longitudinal studies investigating the relationship between the COVID-19 lockdowns and mental health. It concluded that lockdowns do not have uniform detrimental effects on mental health and that most people are psychologically resilient to their effects. Contextual factors, methodological differences, and mental health outcomes could explain several differences.

Research on the psychological reactions to previous epidemics suggests that various psychological vulnerability factors may play a role in inducing coronaphobia, including individual difference variables such as the intolerance to uncertainty, perceived vulnerability to diseases, and anxiety (worry) proneness [7]. While lockdowns and social distancing are well recognised as effective public health measures to reduce the spread of the disease, which many countries have therefore implemented, studies related to other previous restrictions in response to epidemics suggest long-lasting negative effects on people’s mental health. Thus, people experienced increased levels of anxiety, depression, and symptoms of post-traumatic stress reactions in the long term after being released from the lockdowns [8,9,10]. Unfortunately, the effects of the stress reactions on mental health are difficult to determine, as studies show that they affect different social groups differently [11,12,13,14].

Regarding the COVID-19 pandemic, several studies have reported a possible negative relationship between depression, anxiety, PTSD, and age [15,16]. Nwachukwu et al. [17] indicate that both the prevalence rates and mean scores for stress, anxiety, and depression on standardised scales were highest amongst those under 25 years of age and lowest amongst those aged over 60 years. The loss of social connections with friends and exposure to more information about the virus via social media may increase the vulnerability of younger adults to mental distress [18,19,20]. 

National and international surveys have revealed that one in two young adults (aged 18–29 years) are subject to depression and anxiety and one in six are probably affected [21]. These results are in line with many studies on the current pandemic revealing higher psychological distress and depressive symptoms, particularly among young adults [22,23].

University students appear to be particularly susceptible to mental health problems because they are under pressure to perform academically, which in turn disrupts their academic routine and they end up questioning their career trajectory [24,25]. While there is increasing empirical evidence to the consequences of the pandemic on youth adults’ mental health, a better understanding of the impact of COVID-19 on vulnerable groups such as university students requires extensive investigation. Currently, only limited information is available about the psychological impact of lockdowns on college students and the risks it poses by exacerbating their isolation and psychological vulnerability [26,27].

Compared with other groups, students showed a much higher prevalence of self-reported mental health symptoms, including a high level of perceived stress, severe depression, and a high level of anxiety than non-students [28,29]. They were most affected by social isolation and the concern over personal health and that of family members and friends [30,31,32]. When lockdowns were in place, the relationship between perceived stress, emotional distress, and boredom proneness was less explored. Findings showed that boredom proneness mediated the relationship between perceived stress and emotional distress [33,34,35].

The COVID-19 preventive measures affected all domains of social and economic life as well as the education sector. Such restrictions have affected higher education globally, especially the teaching and learning processes, and universities and colleges have been facing unexpected challenges. More than 160 countries implemented nationwide closures, impacting over 87% of the world’s student population. Universities’ closure, isolation, loss of daily routine, and reduced interaction and social contact with teachers and classmates have similarly affected students’ emotional health [18,26].

Before the lockdowns were first imposed, most university students shifted from their usual residences or campuses to their homes. The relocation process during the lockdowns had a significant psychological impact, indicating an increased level of anxiety, particularly a high level of stress, among students who did not shift residences compared with those who did [27].

Research over the past decade has shown a steady increase in the prevalence of depression, anxiety, and suicidality among college students worldwide; even in regular academic periods, psychological distress and mental disorders are common, particularly when they are temporarily away from their schools [36,37,38,39,40,41]. It is acknowledged in the literature [42] that stressors that exceed an individual’s ability to develop coping strategies can lead either to somatic or mental illnesses, or the exacerbation of previous problems, such as depressive symptoms and anxiety. Perceived stress is a subjective experience, as it depends on how each individual interprets both the causes of stress and their magnitude as well as their belief in their ability to cope with stressors, self-esteem and self-efficacy [43]. Therefore, the reactions to stress, specifically stress management, depend on cognition, more precisely the meanings attributed to the two aforementioned aspects. When the cognition is biased, the active coping strategies will be less used, activating defensive mechanisms [44]. Moreover, studies have highlighted that prolonged exposure to stressors affects mental health and the academic performance of university students [45].

High rates of mental health problems and low treatment utilisation are major concerns on students in all types of higher education institutions. The psychological effects of pandemic and lockdowns imposed can exacerbate university students’ mental health issues, and students with pre-existing mental health issues would need extra support during the lockdowns. The general health status, spending extensive time on screens, and knowing someone infected with COVID-19 were considered risk factors for higher levels of negative psychological impact [46].

The influence of possible stressors on students’ mental health, the fear of infection in general, and the inadequate supplies of basic infection-control materials were considered predictors of increased negative psychological impact [47]. Students of all academic levels (high school, undergraduate, and postgraduate) experienced a wide range of negative feelings, including anxiety, depression, tiredness, stress and being overwhelmed, and they identified the lockdowns’ circumstances as unpleasant or very unpleasant [48,49,50].

The sudden disruption of their activities, the switch to and focus on remote education through online learning, social distancing, limited interaction with others, fear of infection, frustration and boredom, and inadequate supplies and information were among the significant stressors during quarantine and accounted for their anxiety and stress [51,52,53].

The rapid spread of the novel coronavirus also affected Romania in the early months of 2020, when the reported cases increased to 1000 within a month since the authorities announced the first case on 26 February 2020. Restrictive measures were implemented at the national and regional level, with the first quarantine measures carried out through a military ordinance in the northwest part of the country intended for 200,000 people [54].

The rigorous measures of the two major lockdowns, replaced with softer measures under a state of alert, were declared at the national level, significantly impacting all activities, including those pertaining to education. Transition to online learning, within a short period, was achieved in most leading universities in Romania as e-learning platforms, although not regularly used, were already established. After solving inherent difficulties related to the lack of equipment, low Internet coverage in students’ residences, and technology issues, most students were able to participate in all activities [55]. Fortunately, with a mere average of 200 cases confirmed per day and a reduced incidence rate, Romania was only moderately affected by the first wave of the disease.

To contain the spread of the pandemic, strict ‘stay at home’ orders were in place between 24 March and 15 May 2020, during the state of emergency, declared by two presidential decrees [56,57]. These measures concerned 377,370 undergraduate students enrolled for the academic year 2019–2020 in 49 public universities across the country.

Following the recommendations of public health authorities, the University of Bucharest suspended didactic activities on 11 March 2020 and 22,799 undergraduate students enrolled in different study programmes continued their learning online until the end of the academic year. Most students left Bucharest to return to their homes during the lockdown and the following period, living with their parents and families, which reduced the negative impact of the lockdowns [27].

The sudden move to remote learning and social distancing presents challenges from a mental health perspective, including feelings of isolation and loneliness due to a lack of face-to-face collaboration, reduced physical and social interaction, and a significant amount of time being spent in front of screens. Unexpected changes in the mode of teaching and the spreading information about the increase in the number of cases could have adverse psychological effects.

Studies related to the SARS-CoV-2 outbreak on students’ mental health in Romania are limited [58]. While many studies analyse the general population’s perception of the pandemic [59], different population groups’ attitudes and responses to the restrictive measures [60,61], and the influence of the pandemic on the economy [62] or quality of life [63], few studies have focused on its psychological impact [64].

## 2. Materials and Methods

### 2.1. Participants

The initial sample consisted of 805 Romanian undergraduate students, from the University of Bucharest, aged 18–26, and enrolled in a university degree programme, in their first, second or third year of study. In terms of attrition rate, 53 were removed due their incomplete answers, resulting in a sample of 722 undergraduate students (M = 21.1 years; SD = ± 1.73; male 34.2%, M = 21.1 years SD = ± 1.71; 65.8% female; M = 21.2 years; SD = ± 1.79).

### 2.2. Measures

Sociodemographic variables included age, gender, place of residence during the lockdowns (Bucharest, other urban areas, or rural areas), living area, and density (number of people living in the house). According to the students’ place of residence during the lockdown, the distribution of students was 71.6% in urban areas, out of which 32.1% were living in Bucharest, and 28.4% students were living in rural areas. More information about sociodemographic variables’ distribution by the stress level can be found in Table 1.

The stress subscale from the *Depression, Anxiety and Stress Scale*, DASS-21, Romanian version [65,66,67] was used to measure the presence of negative emotional symptoms related to stress. The DASS-21 consists of three self-report scales, which measure depression, anxiety, and stress. When completing the DASS-21 questionnaire, the respondents had to mention any presence of a negative emotional symptom over the previous week. The stress subscale focuses on persistent arousal and tension. Each item, rated on a 4-point Likert scale, ranges from 0 to 3 as follows: 0 = did not apply to me at all; 1 = applied to me to some degree or some of the time; 2 = applied to me to a considerable degree or a good part of the time and 3 = applied to me very much or most of the time). A sample item for the stress subscale includes the following: ‘I found it hard to wind down’, and ‘I found it difficult to work up the initiative to do things‘. A higher score indicates high levels of stress. More specifically, the total stress subscale score was divided into normal (0–10), mild stress (11–18), moderate stress (19–26), severe stress (27–34), and extremely severe stress (35–42). [66] The scale has good internal reliability; Cronbach’s α for the entire scale obtained in the present study was 0.92; 95% CI [0.91, 0.93] and the Stress Scale was 0.84; 95% CI [0.82, 0.86].

*The Boredom Proneness Scale–Short Form* (BPS-SR) [68] measures the propensity to boredom in terms of trait not state. According to the creators of this scale, boredom as a trait reflects a failure to regulate oneself, which would result in effective engagement. The BPS-SR is an eight-item scale whose responses are rated on a 7-point Likert-type scale ranging from 1 to 7 (1 = strongly disagree; 7 = strongly agree). The possible scores range from 1 to 56. High scores indicate a higher tendency to experience boredom. A sample item for BPS-SR includes the following: ‘In most situations, it is hard for me to find something to do or watch to keep me interested’; ‘Unless I am doing something exciting, even dangerous, I feel half-dead and dull’. Cronbach’s α obtained in the present study was 0.81; 95% CI [0.78, 0.83].

*The Missing Daily Social Interactions’ Scale* (DSIMS) is a self-report scale developed in the current research to measure to what extent the students felt that they lacked daily social interactions during the pandemic. It is a four-item, one-factor scale. Responses were rated based on a 4-point Likert scale ranging from 0 to 3 (0 = not at all; 1 = a little bit; 2 = a lot; and 3 = very much). The possible scores range from 0 to 20. It was considered that the higher the total score, the higher the level of missing daily social interactions (DSIM). The scale has good internal reliability, with Cronbach α = 0.83 and 95% CI [0.82, 0.86]. As detailed below (see Exploratory factor analysis-EFA and Confirmatory factor analysis-CFA presented in the Results section), the DSIMS has superior psychometric properties, which are suitable for measuring students’ perception of missing daily social interactions during the pandemic. 

*Pandemic-related questions*. To examine whether pandemic-related factors had any influence on participant’s behaviour, they were asked (1) whether they spent more time on phone conversations than usual; and (2) how often they watched the news about the pandemic on TV or social media. The answers for the first item were dichotomous (i.e., it gave ‘yes’ or ‘no’ responses). The second item was rated on a 3-point Likert scale ranging from 1 to 3 (1 = less often; 2 = the same as before; and 3 = more often).

### 2.3. Procedure

Using a cross-sectional design, more precisely a booklet including various scales (as mentioned previously), we collected information to determine the effects of the COVID-19 lockdowns and pandemic restrictive measures taken by public health authorities on the university students’ mental health status at six months after their implementation. The research was carried out utilising a convenience sampling method. Participants were recruited from students enrolled at the University of Bucharest’s various undergraduate study programmes in the social sciences area, which were engaged in different practical activities at the end of the academic year. The data were collected online. The online survey has been shared via e-mail and WhatsApp by the academic tutors of different years of study from 10 to 30 September 2020.Participants were informed about the purpose of the study. Informed consent was obtained electronically before data collection. All participants voluntarily gave their informed consent to participate in the study. The students were asked to fill an online questionnaire. The questionnaires were anonymous to ensure the confidentiality and reliability of data. All procedures complied with the ethical standards of the responsible committee on human experimentation, and the study adhered to the tenets of the Declaration of Helsinki, 1975, as revised in 2000. Further, approval was obtained from the ethics committee of the University of Bucharest (Reg. No. CEC: 063/27.04.2020).

### 2.4. Present Research in the Context

Based on previous studies [69] that emphasised the increase of psychological distress among the general population during the COVID-19 pandemic, the current study analyses stress as an indicator of mental health among university students. To our knowledge, this is the first study that explores the effects of lockdowns and restrictive public health measures on Romanian students’ mental health. It is a retrospective study aiming to evaluate the psychological effects of these unprecedented measures on undergraduate students from the University of Bucharest six months after the lockdowns were put in place. 

We started research with the general assumption that quarantine and self-isolation negatively affect students’ mental health during national lockdowns and the following period. Our research aimed to investigate the mediating role of following news about the pandemic and more time spent on phone conversations (TSPC) in the relationships between missing daily social interactions (DSIM) and boredom proneness on the one side and the perceived stress among university students during and after the lockdowns until September 2020 on the other side.

#### Research Hypotheses

Drawing on the literature that has emphasised that periods of pandemic elicit adverse psychological outcomes [70,71,72,73], we presumed the following:

**Hypothesis** **1:**  *University students’ perceived stress is positively related to missing daily social interactions (DSIM), boredom proneness, following news about the pandemic, and spending more time on phone conversations than usual (TSPC)*.

Considering (1) the role of the negative news about the pandemic on social media in influencing users’ feelings, recall, and information seeking [74], (2) the theory of negativity bias [44,75], which highlights that negative information is more salient than positive information within the context of social information processing and interpretation of meanings assigned to emotions, and (3) the low arousal contexts during the pandemic (due to social isolation, social distancing, limiting or even banning meetings in large groups, online education), we supposed the following:

**Hypothesis** **2:**  *Missing daily social interactions (DSIM), boredom proneness, following news about the pandemic, and more time spent on phone conversations (TSPC) are predictors of university students’ perceived stress*.

Taking into account that there has not been any research on direct and indirect effects of boredom proneness, particularly DSIM on university students’ perceived stress during the COVID-19 lockdowns, we posed the following research question:

**Research** **Question:**  *Does the proposed mediation model including DSIM and boredom proneness as exogenous variables, respectively following news about the pandemic, more TSPC, and perceived stress as endogenous variables fit well with the data?*

Relevant literature [58] recognises that fear increases during pandemics, and individuals’ information needs about what is happening increase, and therefore, they show a great tendency to seek out more information online [75]. In addition, during the pandemic, within the context of the limitations of personal interactions and the possibilities of spending free time in large groups, it seems plausible that people have experienced the feeling of sub-stimulation more frequently. According to Vodanovich et al. [76], boredom proneness is a personality trait that reflects the tendency to have relatively low arousal and dissatisfaction, not within monotonous situational contexts but in inadequately stimulating situations. Individuals with high boredom proneness found it more challenging to adapt to situations characterised by low arousal. Furthermore, immersing oneself in the negativity of news about a threatening situation is conducive to increasing stress. 

Thus, we supposed the following:

**Hypothesis** **3.1:**  *Following news about the pandemic mediates the relationship between boredom proneness and perceived stress*.

With measures resulting in social isolation to prevent the spread of the virus, the time individuals spend on the Internet and technological devices not only to relieve their boredom but also to stay up to date with the news on the pandemic have increased. Therefore, we assumed the following:

**Hypothesis** **3.2:**  *More TSPC mediates the relationship between boredom proneness and perceived stress*.

Considering that one of the inherent reactions to a threatening situation is trying to cope with it using various strategies, it seems plausible that one of these behavioural responses could be more TSPC. However, in the context of social isolation during the pandemic, we believe that TSPC does not help with coping because it cannot substitute face-to-face interaction, and additionally, it does not favour finding solutions. It is accepted in the literature that smartphone addiction symptoms are significantly and negatively related to the level of face-to-face communication and positively related to the absence. A greater amount of smartphone use suggests a lower level of face-to-face communication [77]. Therefore, we presumed the following:

**Hypothesis** **3.3:**  *More TSPC has a mediating role in the relationship between the habit of following news about the pandemic and perceived stress*.

Based on various studies on the psychological outcomes of social isolation during national lockdowns [58,71], we hypothesised as follows:

**Hypothesis** **3.4:**  *DSIM indirectly impacts perceived stress through more TSPC*.

According to Valentino et al. (2008) [78], fears generated due to threatening situations boost the tendency to seek out more information to manage them. In addition, keeping up with negative news could increase the effect of negative mood, related to boredom proneness [76], on perceived stress during lockdowns. Thus, we assumed the following:

**Hypothesis** **3.5:**  *Following news about the pandemic has a mediating role in the relationship between boredom proneness and TSPC*.

### 2.5. Statistical Analysis 

Statistical Package for Social Sciences (SPSS 23) [IBM Statistics, New York, NY, USA] and Analysis of Moment Structure (AMOS 23) [IBM Statistics, New York, NY, USA] were used to analyse the data. Reliability of the scales and univariate normality distribution were testified. Exploratory factor analysis, employing a principal axis extraction method and confirmatory factor analyses, was performed using AMOS to verify the construct validity of the missing daily social interactions’ scale. The multivariate normality distribution of variables was measured using Mardia’s Multivariate Normality Test. 

The various paths of the mediation model were tested using path analysis based on maximum standard likelihood estimation (MLE) with bootstrapping method (with 5000 bootstrapped samples) to deal with the multivariate nonnormality. Model fit was verified by absolute indices: GFI (goodness-of-fit index), AGFI (adjusted goodness-of-fit-index), model chi-square value, *p*-value, df, Cmin/df, RMSEA (root mean square error of approximation), CFI (Bentler comparative fit index), NFI (Bentler–Bonnet normed fit index), IFI (Bollen incremental fit index), and TLI (Tucker–Lewis fit index). According to Hu et al. (1999) [79], the cutoff criteria for comparative fit indexes is ≥0.95, for RMSEA < 0.06, for RMR smaller the better, 0 indicating a perfect fit, for GFI ≥ 0.95 and SRMR < 0.08.

## 3. Results

Descriptive statistics (as shown in Table 2) proved that research variables have not had a substantial departure from univariate normality, considering that skewness values were less than 2, and the kurtosis values less than 7, as recommended by West et al. (1995) [80]. The results showed high scores in terms of DSIM and the following of the news about the pandemic. Moderate levels of boredom proneness and TSPC were also found. In terms of stress, a slightly moderate level was highlighted.

Further, the EFA and CFA were computed to check the construct validity of the proposed scale, i.e., the DSIMS. The findings obtained in the EFA revealed one single factor structure. The Kaiser–Meyer–Olkin measure of sampling adequacy was 0.79, which was above the commonly recommended value of 0.6, and Bartlett’s test of sphericity was significant (χ2(6) = 1320.92, *p* < 0.001). The diagonals of the anti-image correlation matrix were also all over 0.5 (ranging from 0.75 to 0.89). The communalities were above 0.3 (ranging from 0.42 to 0.77), confirming that each item shared some common variance with the other items.

The results obtained in the CFA provided evidence for the excellent fit of the proposed model (i.e., 4-item one factor structure) to the observed data. More precisely, fit indices were well above the cutoff criteria: χ2 (1) = 0.525, CMIN/df = 0.525, *p* = 0.469; CFI = 0.999, TLI = 1.002, GFI = 0.999, AGFI = 0.996, RMSEA = 0.01, 90% CI [0.00, 0.08], RMR = 0.004, *p*close = 0.751. In addition, it had good factor loading values for all items (ranging from 0.65 to 0.88). Given these overall indicators, CFA proved that the DSIMS is suitable to assess students’ perception on daily social interactions in the context of social isolation during the pandemic.

The results obtained in the correlation analysis confirmed the first hypothesis. We found a positive association between perceived stress and all the other research variables (Table 3). In particular, a moderate relationship between stress and boredom was obtained. Small but significant correlations between stress and (1) DSIM, (2) following news about the pandemic, and (3) more TSPC were highlighted (Table 3).

As assumed in the H2, significant regression equations were found. The results showed that DSIM (β = 0.28, *t* (720) = 7.99, *p* < 0.001), boredom proneness (β = 0.41, *t* (720) = 12.14, *p* < 0.001), following news about pandemic (β = 0.14, *t* (720) = 3.80, *p* < 0.001), and more TSPC (β = 0.29, *t* (720) = 8.34, *p* < 0.001) predicted university students’ perceived stress.

**Figure 1:**
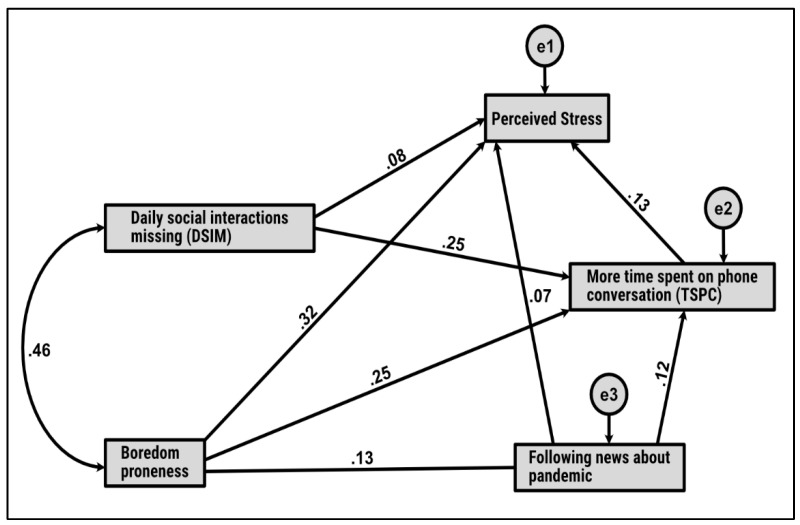
Mediation model: The impact of DSIM and boredom proneness on perceived stress, more TSPC, following the news about the pandemic, and perceived stress; e1, e2, e3-residual variables.

To answer the research question related to the mediation model, a path analysis was performed (Figure 1).

All direct and indirect effects of DSIM and boredom proneness on university students’ perceived stress were computed (Figure 1). Three criteria recommended by Schumacker and Lomax (2004) [81] were applied to verify the statistical significance of the proposed model: (1) non-statistically significant chi-square test; (2) the statistical significance of each parameter estimates; (3) the extent and direction of the parameter estimates to show that they are consistent with the substantive theory. The results confirmed that all criteria were met. The absolute fit index (χ2 = 2.34, df = 1) and non-significant *p*-value (*p* = 0.126) shed light on a very good fit to the data, with: GFI = 0.999, AGFI = 0.981, PGFI = 0.284, CFI = 0.997, TLI = 0.974, RMSEA = 0.04, 90% CI [0.01, 0.11], *p*close = 0.427, RMR = 0.056 and SRMR = 0.013.

Testing the various mediation models included in the path analysis validated all hypotheses. First, H3.1 proved that the indirect effect of boredom proneness on perceived stress by following the news about the pandemic was statistically significant (β = 0.03; [0.02, 0.05]; *p* < 0.001). These findings provided evidence for a partial mediation because the direct effect (β = 0.31; [0.28, 0.33]; *p* < 0.001) remained statistically significant after controlling for the mediator, namely following the news about the pandemic.

Second, as assumed in H3.2, more TSPC mediated the relationship between boredom proneness and perceived stress. In addition to the aforementioned direct effect, an indirect effect was obtained (β = 0.02; [0.01, 0.04]; *p* < 0.001).

Third, when testing H3.3, a partial mediation was found, i.e., following the news about the pandemic had a direct effect on perceived stress (β = 0.07; [0.05, 0.08]; *p* < 0.001) and an indirect effect through more TSPC (β = 0.02; [0.01, 0.05]; *p* < 0.001). Fourth, H3.4 was validated because a partial mediation was observed.

More specifically, more TSPC mediated the relationship between DSIM and perceived stress. Thus, a statistically significant direct effect (β = 0.08; [0.07, 0.09]; *p* < 0.001) and indirect effect (β = 0.04; [0.02, 0.07]; *p* < 0.001) were obtained. Fifth, considering the last assumed mediation in H3.5, the findings provided evidence for a significant direct effect (β = 0.24; [0.19, 0.27]; *p* < 0.001) and an indirect effect (β = 0.02; [0.01, 0.04]; *p* < 0.001) of boredom proneness on more TSPC from following the news about the pandemic.

Furthermore, we analysed gender differences in terms of all research variables to find that female students have significantly higher level of stress (M = 14, SD = 5.98) than male students (M = 11.25, SD = 5.42; *t* (720) = −3.74, *p* = 0.001. Female students also perceived to a greater extent (M = 9.05, SD = 3.44) than males (M = 8, SD = 3.29) regarding the DSIM (*t* (720) = −4.03, *p* = 0.001). A similar pattern was obtained in the case of TSPC, which is where female students (M = 1.86, SD = 1.06) spent more time than male students (M = 1.54, SD = 1.08) on phone conversations (*t* (720) = −3.80, *p* = 0.001).

## 4. Discussion

The first finding of our study revealed that students with high proneness to boredom and a high level of DSIM who spend more time on phone conversations and follow the news about the pandemic have a higher score on perceived stress. H1 has therefore been proven. These findings align with previous research [82], highlighting that fear triggers information-seeking. It seems plausible that the information-seeking about the pandemic during lockdowns would also have taken place in the context of more TSPC, especially since face-to-face interactions were limited. In addition, the correlation between perceived stress and all research variables is in line with the previous findings mentioned in a meta-analysis [83] that focused on the increasing global prevalence of mental health issues among the general population during the COVID-19 pandemic, such as stress, depression, and anxiety.

The second finding of this research highlighted some predictors of perceived stress, specifically personality traits (boredom proneness), needs (daily social interactions), and behavioural responses (more TSPC). These results support H2. Similar to previous research [84], we provided evidence that proves boredom proneness is conducive to stress experience.

In terms of the predictive role of the following of news about the pandemic on perceived stress, it is acknowledged in the literature on the neurocircuitry of fear [85] that repeated activation of the brain regions is responsible for negative emotions, such as fear; the perception of alarming content broadcast on the news accentuates alertness, insecurity, stress, and anxiety. Alarming content propagated during the lockdowns pertained to worrying statistics, medical staff overwhelmed by the severity of the situation, absence of vaccines, and daily rise in the death rate. All this created a state of alertness, and more TSPC did not alleviate stress but increased it. In addition, there are studies [86,87] that have shown that in the context of the COVID-19 pandemic, there were effects on the mental health condition of students, in terms of burnout syndrome symptoms, due to the long periods spent in front of computer screens and the hardships resulting from online learning.

The third finding of this study emphasised that the direct and indirect effects of DSIM, namely boredom proneness, on university students’ perceived stress through various mediators. These findings supported all sub-hypotheses posited to testify the mediation model. More specifically, we proved the mediating role of TSPC in the relationship between following the news about the pandemic and perceived stress. This suggests that perceived stress increases among students with a high tendency to follow the news about pandemic increases, while the high level of TSPC increases stress even more.

We can explain this pattern, considering that phone conversations do not help manage one’s issues, including worries, fears, and the absence of social interactions. In addition, more TSPC cannot compensate for face-to-face communication. It probably prolonged the state of alertness induced by the alarming content in the news. When individuals focus on negative emotions and threats without finding solutions, the phenomenon of ruminative thinking may occur. Additionally, our findings emphasised that following the news on TV or social media to keep up with the events related to the pandemic plays a mediating role in the relationship between boredom and perceived stress.

These results align with research on negativity bias which refers to a phenomenon whereby humans tend to focus more on negative information than on positive information in their feelings, judgments, and information-processing tasks [75]. Additionally, salient negative information tends to elicit stronger emotional, cognitive, and behavioural responses than neutral or positive information [88]. In other words, the consciousness of negative events increases perceived stress.

We also testified to the mediating role of TSPC in the relationship between DSIM and perceived stress. This result matches the research that proposed that increased restrictive measures in the context of the COVID-19 lockdowns have caused individuals to change their daily routines drastically and has also resulted in increased fear and stress among many individuals [89].

Regarding the partial mediation of following the news about the pandemic in the relationship between boredom proneness and TSPC, we can conclude that TSPC increases among students with high boredom proneness, while among students with a high tendency to follow the news about the pandemic, it increases even more. We can interpret these results from the perspective of Fredrickson’s theory [90], according to which negativity—created by boredom and unpleasant and anxious contents broadcast on the news—narrows the ability to find solutions. Similarly, Ohman et al. (2000) [91] showed that fear involves uncertainty about one’s ability to withstand or handle a given threat. That is why we found out that the phone conversations did not have a compensatory effect for those students characterised by higher boredom proneness and accentuated the perceived stress. These results are parallel to those [92] that have already discussed the link between problematic phone use and maladaptive responses to environmental stressors.

In terms of gender differences, our findings revealed that female students experienced higher stress levels than male students. These results correlate with the previous studies that emphasised that the formative period for young adults can have repercussions for their psychological and physical health [93], and male students face less stress than their counterparts [94]. We also found gender differences in DSIM; this is an expected pattern, considering the study [95] on the relationship between affiliation motivation and daily experience that suggested that girls spent more time with friends and less time alone than boys. Therefore, it does not seem surprising that in our research, females felt more affected due to missing daily social interactions (DSIM). Consequently, they spent much more time on phone conversations than males during social restrictions related to the COVID-19 pandemic.

### Study Limitations 

We measured the level of anxiety, stress, and depression six months after the lockdowns. Therefore, our study is retrospective rather than longitudinal, which decreases our ability to say with confidence that COVID-19 restrictive measures, including lockdowns, caused the reported impacts. However, we are confident that the findings are attributable to the pandemic, given our survey prompts/inquiry requests.

Due to the lack of pre-pandemic assessments of the anxiety, stress, and depression, we could not compare pre-existing mental health problems that were subsequently amplified by the lockdown. The higher number of female students in the sample structure was likely the result of the demographic composition of the departments to which students belong. This study was based in the University of Bucharest, the largest university in Romania that has students from all over the country. While the structure of their programmes and restrictive measures taken were the same, during the lockdowns, most of the students returned to their homes where they faced different situations in terms of the increase in the number of cases and deaths. Further, studies could help assess the progression or even a potential rebound effect of the psychological manifestations once the imminent threat of COVID-19 subsides.

To overcome the disadvantages of convenience sampling, future studies based on sampling techniques such as random, stratified, or cluster sampling that yield generalisable estimates are needed. Our results are not necessarily generalisable to a population with other characteristics than those specified in the research, namely university students, social sciences profile, and rural/urban place of residence.

It is desirable for greater generalizability that future studies examine other sociodemographic categories, apart from university students at social sciences profile. In addition, it is necessary to expand our sample by including various specialisations, like STEM. Thus, another shortcoming, gender imbalance, can be overcome (in social sciences, many more females than males are enrolled). It would be interesting to examine whether the same psychological mechanisms mediating the relationship between DSIM and boredom proneness on the one side and perceived stress on the other come into operation with other cohorts.

## 5. Conclusions

Even though the national lockdowns and many restrictive measures have now been lifted, the pandemic will continue to significantly affect university students in the coming months. The specific nature of the sample may also restrict the generalisation of conclusions. The findings nonetheless suggest that the public health strategies used to combat COVID-19 and specific psychological interventions may correlate with mental health indicators.

We discussed in the current research that DSIM and boredom proneness are directly associated with university students’ perceived stress and indirectly through more TSPC than usual. Additionally, our findings emphasised that keeping up with the events related to the pandemic by following the news has a mediating role in the relationship between boredom proneness and perceived stress. The behaviours investigated, i.e., the habit of spending more time on phone conversations and keeping up with events by following the news, did not have a buffer effect on stress and rather contributed to its increase.

Therefore, university students need to receive support through programmes focused on increasing emotional self-regulation skills and developing coping strategies to maintain mental health even in adverse contexts.

Student’s mental health is a public health issue that has become challenging to address, especially during the pandemic. Although most universities re-started courses in September 2020, the rules of social distancing have required the use of distance or hybrid learning, for which students and teachers are less prepared. These are contributing to a weakening of protective factors including daily routine and social interactions that further support good mental health. The lack of a regular schedule, technology issues, and the increased flow of information to students can lead to students dropping out and academic failure. Thus, public authorities and universities need to pay more attention to the effects; insufficient efforts to recognise and address these issues could have long-term consequences on their health and education. Governments should consider investing in mental health, expanding service availability, and increasing capacity by introducing new forms of mental health support for youth (hotline platforms, phone lines, youth support centres, teleconsultations, awareness campaigns). A mental-health-in-all policies approach that considers the interlinkages of mental health with other policy areas is required [96].

## Figures and Tables

**Table 1:** Sociodemographic variables distribution depending on level of perceived stress.

Sociodemographic Variables		Level of Stress					Total
		Normal (0–10)	MildStress (11–18)	Moderate Stress (19–26)	Severe Stress (27–34)	Extremely Severe Stress (35–42)	
Gender	Male	140 (19.4%)	44 (6.1%)	35 (4.8%)	24 (3.3%)	4 (0.6%)	247 (34.2%)
	Female	224 (31.0%)	90 (12.5%)	71 (9.8%)	76 (10.5%)	14 (1.9%)	475 (65.8%)
Place of residence	Urban	267 (37.0%)	90 (12.5%)	81 (11.2%)	68 (9.4%)	11 (1.5%)	517 (71.6%)
	Rural	97 (13.4%)	44 (6.1%)	25 (3.5%)	32 (4.4%)	7 (1.0%)	205 (28.4%)
Living during lockdown	Urban blocks of flats	183 (25.3%)	54 (7.5%)	52 (7.2%)	42 (5.8%)	8 (1.1%)	339 (47%)
	Rural block of flats	1 (0.1%)	4 (0.6%)	1 (0.1%)	2 (0.3%)	1 (0.1%)	9 (1.2%)
	Urban detached house	72 (10.0%)	32 (4.4%)	26 (3.6%)	19 (2.6%)	3 (0.4%)	152 (21,1%)
	Rural detached house	108 (15.0%)	44 (6.1%)	27 (3.7%)	37 (5.1%)	6 (0.8%)	222 (30.7%)
Density	1 person	9 (1.2%)	9 (1.2%)	5 (0.7%)	3 (0.4%)	2 (0.3%)	28 (3.9%)
	2 people	86 (11.9%)	21 (2.9%)	15 (2.1%)	19 (2.6%)	3 (0.4%)	144 (19.9%)
	3 people	141 (19.5%)	44 (6.1%)	36 (5.0%)	28 (3.9%)	6 (0.8%)	255 (35.3%)
	>3 people	128 (17.7%)	60 (8.3%)	50 (6.9%)	50 (6.9%)	7 (1.0%)	295 (40.9%)

**Table 2:** Descriptive statistics of research variables—mean, SD, skewness, and kurtosis.

Research Variables	Mean	*SD*	Skewness		Kurtosis	
			Statistic	Std. Error	Statistic	Std. Error
Perceived stress	13.14	6.17	0.05	0.091	−0.98	0.182
Boredom proneness	32.02	7.56	−0.06	0.091	0.96	0.182
DSIM	8.69	3.38	−0.79	0.091	0.40	0.182
TSPC	1.75	1.08	−0.24	0.091	−1.25	0.182
Following news about pandemic	2.36	0.88	−1.19	0.091	0.41	0.182

Notes: DSIM = Missing Daily Social Interactions; TSPC = Time spent on phone conversations.

**Table 3:** Correlation matrix of research variables: perceived stress, boredom proneness, DSIM, following news about the pandemic, and more TSPC.

Variables	1	2	3	4	5
Perceived stress	-				
Boredom proneness	0.412 **	-			
DSIM	0.286 **	0.463 **	-		
Following news about pandemic	0.140 **	0.128 **	0.109 **	-	
More TSPC	0.297 **	0.381 **	0.381 **	0.176 **	-

Notes: DSIM-Missing Daily Social Interactions; TSPC-Time spent on phone conversations. ** *p* < 0.01.
